# Non invasive neurostimulation

**DOI:** 10.1186/1129-2377-16-S1-A18

**Published:** 2015-09-28

**Authors:** Licia Grazzi

**Affiliations:** U.O. Neurologia III, Fondazione IRCCS Istituto Neurologico C. Besta, Milan, Italy

Neurostimulation has been used in pain management for a long time. Several neuromodulatory surgical techniques were developed to manage headaches unresponsive to medical treatment, in particular for preventive treatment. Recently, novel non invasive neurostimulation approaches have been used successfully for different forms of headache: they represent a promising opportunity in headache management useful for acute and preventive treatment of migraine. Among the different non invasive approaches, transcutaneous supraorbital electrostimulation device (Cefaly), transcutaneous magnetic stimulation (TMS), transcranial electrical stimulation (t DCS), and non-invasive vagus nerve stimulation device (n VNS) have shown efficacy in a consistent number of pilot studies (lack of placebo condition). Vagus Nerve Stimulation (VNS) was a well known surgical procedure approved for treatment of refractory epilepsy and depression, with a peripheral mechanism of action. It has been used in selected intractable migraine cases if associated with comorbid depression, with promising results. A hand-held patient controlled non-invasive vagus nerve stimulation device (Gammacore) has been developed to treat migraine attack and has contributed to an easier treatment of migraine episodes. Goadsby et al reported significant results from treatment of migraine attacks by non-invasive transcutaneous vagal nerve stimulation in a population of 30 patients with a 2-hour pain-free rate of 22%. In our open-label, single arm, multiple attack, 27 patients, 18-65 years old, with a diagnosis of migraine without aura according to the IHS beta 2013 classification, with a high frequency, were enrolled and participated in the study with 112 attacks treated. Treatment consisted of one stimulation, 90-second dose, delivered to the right cervical branch of the vagus nerve. In forty-four attacks (39.2%) 9 resolved within 30 minutes; 50 attacks (44.6%) did not resolve in the first 2 hours, so patients used rescue medication; in 18 attacks (16.2%) there was no complete resolution of pain, but a significant relief in the first 2 hours (40% in the VAS scale) and patients did not use any rescue medication. No adverse events were recorded, the therapy was well tolerated. Non-invasive vagus nerve stimulation (nVNS) offers a novel approach to acute migraine attack. For patients with high frequency of migraine attacks, nVNS may represent a suitable tool to decrease the risk of medication overuse. The variety of non-invasive neuromodulatory approaches has opened new strategies for treatment of patients suffering from drug-refractory forms of headache. Randomized sham controlled studies and larger and multicentre trials are needed in order to determine the optimal dosage and to clarify the mode of action.

